# Supplementary material for: Robustness tests for biomedical foundation models should tailor to specifications
Source: NPJ Digit Med. 2025 Aug 29;8:557. doi: 10.1038/s41746-025-01926-2 (PMC12397224; doi:10.1038/s41746-025-01926-2)
Supplement: Supplementary file 1 — Supplementary Information [file 41746_2025_1926_MOESM1_ESM.pdf]

Supplementary information for Robustness tests for biomedical foundation models should tailor to specifications

R. Patrick Xian[1,2,*], Noah R. Baker[3], Tom David[4], Qiming Cui[5,1], A. Jay Holmgren[6], Stefan Bauer[7], Madhumita Sushil[8], Reza Abbasi-Asl[1,2,9,*]

[1]Department of Neurology, University of California, San Francisco, 1651 4th Street, San Francisco, CA 94158, USA.
[2]Weill Institute for Neurosciences, University of California, San Francisco, 1651 4th Street, San Francisco, CA 94158, USA.
[3]Biological and Medical Informatics Graduate Program, University of California, San Francisco, 550 16th Street, 3rd Floor, San Francisco, CA 94158, USA.
[4]PRISM Eval, 10 Rue de Penthièvre, 75008 Paris, France.
[5]Department of Bioengineering, University of California, Berkeley, 306 Stanley Hall, University Drive, Berkeley, CA 94720, USA.
[6]Division of Clinical Informatics and Digital Transformation, University of California, San Francisco, 10 Koret Way, San Francisco, CA 94117, USA.
[7]School of Computation, Information and Technology, Technical University of Munich & Helmholtz AI, Friedrich-Ludwig-Bauer-Strasse 5, 85748 Garching bei München, Germany.
[8]Bakar Computational Health Sciences Institute, University of California, San Francisco, 490 Illinois Street, San Francisco, CA 94158, USA.
[9]Department of Bioengineering and Therapeutic Sciences, University of California, San Francisco, 1700 4th Street, San Francisco, CA 94143, USA.
[*]Corresponding authors: xrpatrick@gmail.com, reza.abbasiasl@ucsf.edu.

1 Application-specific robustness metrics

The construction of robustness metrics is specific to the use cases because of the data modality and practical requirements involved. Among the three types of robustness metrics, aggregated metrics allow a balanced view of robustness failures and are used as a general assessment. Stratified comparisons across distinct subgroups (e.g. demographics, clinical contexts, temporal shifts, or biological characteristics) offer a comprehensive evaluation of both model performance and ethical alignment. Worst-case metrics set a lower bound on the model performance and are more useful in high-risk settings where the negative effects should be considered fully. In the following, we consider three commonly encountered use cases in biomedical applications and discuss the ways to construct the relevant metrics:

In **diagnostic decision support**, the demographic information is usually directly taken into account. The most common performance metric is accuracy [1]. Evaluations for robustness should include comparison across distinct subgroups such as those defined by age, sex, and race of the patient. The robustness metrics for this task should take into account the disparity between subgroups or use the worst-subgroup accuracy across stratified demographic subgroups.

In **medical image interpretation** or **medical report generation**, the common metrics include semantic overlap such as ROUGE [2], BERTScore [3], and more specialized variants like RadGraph F1 [4], which accounts for relationship and completeness at the level of biomedical named entities in the generated interpretation or report. While medical images do not explicitly encode race information, they do reveal key biological characteristics such as organ morphology, anatomical variation, and biological age and sex. Constructing robustness metrics should account for the model's aggregated performance using out-of-distribution data that include the effects of common image distortion and shifts along biologically relevant covariates that represent anatomical variations.

In **clinical text summarization**, demographic information is often present due to the nature of the text data. The common performance metrics in summarization are semantic overlap and faithfulness (aka. factual correctness). Semantic overlap is like just discussed for the previous task. Quantifying faithfulness requires extraction of the factual component from both the original text and the summarization before comparison [5]. Robustness evaluations for this task should consider text dataset shifts including typos, grammatical errors, variations in narrative style, and documentation practices across institutions. The robust metrics for this task can be an aggregated metric or the difference between stratified subgroups bearing their sensitive subgroup information.

2 Robustness in major AI regulatory frameworks

We provide here more details on the robustness requirements in AI systems from major AI policy recommendations and regulations in the European Union (EU) and United States (US). We quote the corresponding documents wherever needed to illustrate the details presented there.

The **EU AI Act** is the first regulation of AI by a major jurisdiction, the EU. It considers robustness and cybersecurity as related concepts and puts AI models in biomedical and healthcare applications within the high-risk AI systems category [6]. The AI Act delineates various requirements on accuracy,

robustness and cybersecurity together [7] in its Article 15 (https://artificialintelligenceact.eu/article/15/), which will go into force in August 2026. Regarding natural robustness, the AI Act demands that high-risk AI systems "shall be as resilient as possible regarding errors, faults or inconsistencies that may occur within the system or the environment in which the system operates, in particular due to their interaction with natural persons or other systems." Regarding adversarial robustness (considered within the scope of cybersecurity in the AI Act), the AI Act demands that high-risk AI systems "shall be resilient against attempts by unauthorised third parties to alter their use, outputs or performance by exploiting system vulnerabilities."

The **US Federal AI Risk Management Act** promotes the AI risk management framework (https://www.nist.gov/itl/ai-risk-management-framework) and its successors developed by the US National Institute of Standards and Technology (NIST). It is currently a leading framework on the subject issued by a US federal agency [8], but it is yet to be enacted into law as of mid-2025 (https://www.govinfo.gov/app/details/BILLS-118hr6936ih/). The NIST framework is a set of recommendations and it identifies the measurement of risk, tolerance determination of risk, and prioritization of risks as the major challenges in AI risk management. The NIST framework adopts an industry- and use case-agnostic approach and refers to resilience as the counterpart of robustness that also accounts for the resistance to "adversarial use of model or data". The NIST framework encompasses four key processes: map, measure, manage, and govern to be implemented throughout the lifecycle of AI systems. Robustness is featured in the measure process, where the framework mentions that "The AI system to be deployed is demonstrated to be safe, its residual negative risk does not exceed the risk tolerance, and it can fail safely, particularly if made to operate beyond its knowledge limits. Safety metrics reflect system reliability and robustness, real-time monitoring, and response times for AI system failures."

Supplementary data 1

Collected data on robustness tests for biomedical foundation models. The data from over 50 biomedical foundation models include information on the model developers (e.g. institutions), the modality (e.g. language, vision, or both), model capabilities, biomedical domain, and types of robustness tests, along with reference to the respective publication. They are used for creating Figure 1 in the main text.

References

[1] Miller, R. A. in *Diagnostic Decision Support Systems* (ed.Berner, E. S.) *Clinical Decision Support Systems: Theory and Practice* 181–208 (Springer International Publishing, Cham, 2016). URL https://doi.org/10.1007/978-3-319-31913-1_11.

[2] Lin, C.-Y. *ROUGE: A Package for Automatic Evaluation of Summaries.* 74–81 (Association for Computational Linguistics, Barcelona, Spain, 2004). URL https://aclanthology.org/W04-1013/.

[3] Zhang, T., Kishore, V., Wu, F., Weinberger, K. Q. & Artzi, Y. *BERTScore: Evaluating Text Generation with BERT* (2019). URL https://openreview.net/forum?id=SkeHuCVFDr.

[4] Yu, F. *et al.* Evaluating progress in automatic chest X-ray radiology report generation. *Patterns* **4**, 100802 (2023). URL https://www.sciencedirect.com/science/article/pii/S2666389923001575.

[5] Maynez, J., Narayan, S., Bohnet, B. & McDonald, R. *On Faithfulness and Factuality in Abstractive Summarization.* 1906–1919 (Association for Computational Linguistics, Stroudsburg, PA, USA, 2020). URL https://www.aclweb.org/anthology/2020.acl-main.173.

[6] Bellogín, A. *et al.* The EU AI Act and the Wager on Trustworthy AI. *Commun. ACM* **67**, 58–65 (2024). URL https://dl.acm.org/doi/10.1145/3665322.

[7] Nolte, H., Rateike, M. & Finck, M. *Robustness and Cybersecurity in the EU Artificial Intelligence Act.* FAccT '25, 283–295 (Association for Computing Machinery, New York, NY, USA, 2025). URL https://dl.acm.org/doi/10.1145/3715275.3732020.

[8] Rawal, A., Johnson, K. A., Mitchell, C., Walton, M. & Nwankwo, D. *Responsible Artificial Intelligence (RAI) in US Federal Government : Principles, Policies, and Practices.* NeurIPS 2024 Workshop on Regulatable ML (2024). URL https://openreview.net/forum?id=OrwvUD7p5q.
